# Coadministration of lithium and celecoxib reverses manic-like behavior and decreases oxidative stress in a dopaminergic model of mania induced in rats

**DOI:** 10.1038/s41398-019-0637-9

**Published:** 2019-11-13

**Authors:** Samira S. Valvassori, Paula T. Tonin, Gustavo C. Dal-Pont, Roger B. Varela, José Henrique Cararo, Abel Freitas Garcia, Fernanda F. Gava, Samira Menegas, Jair C. Soares, João Quevedo

**Affiliations:** 1 Translational Psychiatry Laboratory, Graduate Program in Health Sciences, University of Southern Santa Catarina (UNESC), Criciúma, Santa Catarina, Brazil; 2 Center of Excellence on Mood Disorders, Department of Psychiatry and Behavioral Sciences, McGovern Medical School, The University of Texas Health Science Center at Houston (UTHealth), Houston, TX USA; 3 Neuroscience Graduate Program, The University of Texas MD Anderson Cancer Center UTHealth Graduate School of Biomedical Sciences, Houston, TX USA; 4 Translational Psychiatry Program, Department of Psychiatry and Behavioral Sciences, McGovern Medical School, The University of Texas Health Science Center at Houston (UTHealth), Houston, TX USA

**Keywords:** Clinical pharmacology, Bipolar disorder, Molecular neuroscience

## Abstract

The present study intends to investigate the effect of lithium (Li) and celecoxib (Cel) coadministration on the behavioral status and oxidative stress parameters in a rat model of mania induced by dextroamphetamine (d-AMPH). Male Wistar rats were treated with d-AMPH or saline (Sal) for 14 days; on the 8th day of treatment, rats received lithium (Li), celecoxib (Cel), Li plus Cel, or water until day 14. Levels of oxidative stress parameters were evaluated in the serum, frontal cortex, and hippocampus. d-AMPH administration induced hyperlocomotion in rats, which was significantly reversed by Li and Cel coadministration. In addition, d-AMPH administration induced damage to proteins and lipids in the frontal cortex and hippocampus of rats. All these impairments were reversed by treatment with Li and/or Cel, in a way dependent on cerebral area and biochemical analysis. Li and Cel coadministration reversed the d-AMPH-induced decrease in catalase activity in cerebral structures. The activity of glutathione peroxidase was decreased in the frontal cortex of animals receiving d-AMPH, and treatment with Li, Cel, or a combination thereof reversed this alteration in this structure. Overall, data indicate hyperlocomotion and alteration in oxidative stress biomarkers in the cerebral structures of rats receiving d-AMPH. Li and Cel coadministration can mitigate these modifications, comprising a potential novel approach for BD therapy.

## Introduction

Bipolar disorder (BD) is a prominent, impactful psychiatric disorder affecting moods, whose lifetime prevalence is ~ 5%^1,2^. The precise pathophysiology of BD remains unclear^3^. However, oxidative stress is linked to this condition, as alterations in the activity of antioxidant enzymes and other oxidative disturbances have been reported in BD patients^4^.

The clinical hallmark of BD is the manic episodes, including euphoria, hyperactivity, insomnia, or hypersexuality, so that animal models able to reproduce mania are one of the experimental resources most used in BD research^5,6^. In this scenario, hyperlocomotion, altered pattern of sleep, and risk behavior were observed in the animal model of mania induced by amphetamine^7–9^. In addition, administration of mood stabilizer drugs, including valproate, can counteract the effect of amphetamine on the locomotion of rats submitted to such dopaminergic model, which supports its validity for the study of the mood disorder^7^. The main limitations of the mania model induced by dextroamphetamine (d-AMPH) in rodents, besides it be a chemically induced model, include mimicking drug abuse symptoms^10,11^ and psychosis-related behaviors (at higher doses)^12–14^.

The BD standard therapy comprises lithium (Li, as carbonate salt) and anticonvulsants, such as valproate^15^. Nevertheless, certain side effects, namely weight gain, polyuria, bradykinesia, sexual dysfunction, and others render the patients affected by the disorder prone to noncompliance to therapy^16,17^. Thus, the design of new therapeutic strategies aiming to minimize or eliminate such undesirable consequences of traditional BD therapy is probably a promising field of research at present.

On the other hand, celecoxib (Cel) was the first anti-inflammatory drug inhibiting the cyclooxygenase-2 enzyme (EC 1.14.99.1) with approval for use by the US Food and Drug Administration (FDA)^18^. Cel was also showed to be useful as adjunct therapy of manic or depressive symptoms^19,20^, in part owing to the finding that a proinflammatory state could be associated with BD^21^. An immune or inflammatory imbalance in the pathophysiology of this mood disorder could constitute a new platform for the development of novel therapeutic approaches^22^.

Therefore, the present study aimed to investigate the effect of Li and Cel coadministration on the behavioral status and oxidative stress parameters in rats submitted to the dopaminergic model of mania induced by d-AMPH.

## Material and methods

### Animals

Adult male Wistar rats (*Rattus norvegicus*; 250–350 g) obtained from the Central Animal House of University of Southern Santa Catarina (UNESC) were used. Rats were caged in groups of five animals and had ad libitum access to water and standard chow. A light/dark cycle (12:12 h; lights on at 7:00 h) at constant temperature (22 ± 1 °C) was maintained in the colony room. All experiments followed The “Principles of Laboratory Animal Care” (National Institutes of Health publication no. 80–23, revised in 1996) and the “EC Directive 86/609/EEC”. This study was only carried out with previous approval by the Local Ethics Committee on Animal Use for Research (protocol # 056/2015–2). All efforts were made to reduce the number of animals and their suffering. Thus, the experiment was carried out with *n* = 12 animals per group.

### Reagents

d-AMPH was purchased from Sigma-Aldrich (Saint Louis, Missouri, United States), whereas Li (carbonate salt) was obtained from Eurofarma (São Paulo city, São Paulo, Brazil) and Cel from Getz Pharma (Karachi, Pakistan). d-AMPH was diluted in saline solution (Sal; NaCl 0.9%) to enable the intraperitoneal (i.p.) administration of the drug. Li and Cel tablets were macerated, and the resulting powder was added to water until form a suspension orally administered to the rats. These suspensions were then kept on a magnetic mixer in order to maintain constant concentrations of the drugs during the administrations.

### Experimental design

Rats received d-AMPH (2 mg per kg body weight [mg/kg]) or Sal during 14 days. Each drug was administered once a day, i.p. From the day 8–14, animals from d-AMPH and Sal groups orally received Li (24 mg/kg), Cel (20 mg/kg), a combination thereof or water. Li was administered twice a day (12:12 h), whereas Cel was only administered once a day. At day 15, rats received an i.p. injection of d-AMPH or Sal and 2 h later were submitted to the behavioral analysis. Thus, the experimental groups in the present study are as follows: (i) Sal + water; (ii) Sal + Li; (iii) Sal + Cel; (iv) Sal + Li + Cel; (v) d-AMPH + water; (vi) d-AMPH + Li; (vii) d-AMPH + Cel; and (viii) d-AMPH + Li + Cel. Dosage of Cel and d-AMPH was following previous studies^7,23^, but the dose of Li was equal to the half of those administered in the study performed by Frey and coworkers^7^.

### Behavioral analysis

Behavioral status was assessed through the open-field test. In this procedure, hyperlocomotion or hyperactivity induced in the animal model is a parameter associated to mania, which characterizes the BD^9^. Open-field test was carried out in a 40 × 60 cm box, whose 50 cm-height-walls are made of brown fiberboard, except the frontal wall, which is made of glass. The floor of the box is divided into nine equal rectangles by black lines. To freely explore the area for at least 5 min, the animals are gently put on the left posterior square. During the procedure, the number of crossings and rearings provides consistent information on the rat behavior and locomotion^24^.

### Sample preparation

After behavioral analysis, rats were killed by decapitation without anesthesia, the skull was opened, and the cerebral content was excised and rapidly dissected on a chilled dish Petri. The frontal cortex and hippocampus were isolated and cleaned from the subcortical structures and white matter. Peripheral blood samples were immediately collected after decapitation and submitted to centrifugation at 10,000 rpm during 15 min aiming to obtain the serum. Until the analyses were performed, all samples were kept frozen at −80 °C.

### Protein carbonyl content

The determination of the carbonyl content was performed in samples of the cerebral structures and serum previously homogenized in sodium phosphate buffer 20 mM and potassium chloride 140 mM (pH 7.4). In brief, the principle of the method is based on the binding of carbonyl groups present in the samples to the 2,4-dinitrophenylhydrazine reagent (Sigma-Aldrich) forming aliphatic hydrazones, whose absorbance values were spectrophotometrically determined at 360 nm wavelength (*λ*), using blanks to each sample. Such information correlates to the carbonyl group content in the samples. Data were expressed as nmol/mL^25^.

### Level of thiobarbituric acid-reactive species

The levels of the thiobarbituric acid-reactive species (TBARS), malondialdehyde (MDA), were measured in samples of the cerebral structures and serum homogenized in chilled PBS buffer (pH 7.4). Homogenates were mixed with trichloroacetic acid (Sigma-Aldrich) 10% aiming to form a precipitate, and sodium sulfate was added to supernatants at a 1:1 v/v ratio. After centrifugation, thiobarbituric acid (Sigma-Aldrich) 0.67% was added to supernatants, at this same v/v ratio. The resulting mixture was submitted to heating for 2 h and then cooled with water at room temperature. Measuring of absorbance values was performed in a spectrophotometer at *λ* = 532 nm. Data were expressed as MDA equivalents nmol/mg protein^26^.

### Catalase enzyme activity

The catalase (CAT; EC 1.11.1.6) activity was evaluated in samples of the cerebral structures and serum according to a standardized procedure^27^. First, Triton 0.1% was added to the sample homogenates, and the resulting mixture was incubated on ice for 15 min. Hydrogen peroxide 30% v/v was then added to potassium phosphate buffer 10 mM, pH 7.0, and the resulting solution was put on a quartz cuvette. SpectraMax microplate spectrophotometer (Molecular Devices, Sunnyvale, California, United States) was calibrated with a blank separately run from phosphate buffer. After sample addition, a decrease in hydrogen peroxide absorbance was measured using the microplate photometer at *λ* = 240 nm during 3 min, with 30 s as an interval between measurements. Data were expressed as U/mg protein.

### Glutathione peroxidase enzyme activity

Glutathione peroxidase (GPx; EC 1.11.1.9) activity in samples of the cerebral structures and serum was measured by Wendel’s method^28^, with tert-butyl hydroperoxide used as substrate. Into each tube containing sample was added potassium phosphate buffer 100 mM, pH 7.0, ethylenediaminetetraacetic acid 1 mM, sodium azide 40 mM, reduced glutathione 100 mM, glutathione reductase (EC 1.6.4.2) 10 U/mL and reduced nicotinamide adenine dinucleotide phosphate 10 mM. The resulting mixture was incubated for 1 min at 25 °C. Thereafter, enzyme reaction was started after the addition of tert-butyl hydroperoxide 10 mM. Blanks were prepared using phosphate buffer instead of the sample. A decrease in absorbance at *λ* = 340 nm was followed during 300 s in a SpectraMax microplate photometer. Calculated GPx activity was expressed as nmol min^−1^ mL^−1^.

### Statistical analysis

The variables were analyzed according to their distribution through Shapiro Wilk’s test for normality. The Levene test assessed the homogeneity of variances among groups. All data were expressed as the mean ± standard error of the mean and were analyzed by three-way analysis of variance (ANOVA) followed by Tukey’s test when *F* value was significant. Statistica 7 software (Dell Software, Round Rock, TX, USA) was used to perform all analyses. The differences between groups were rated as statistically significant at *p* < 0.05. The software used to generate the figure graphs was GraphPad Prism version 5.00 for Windows (GraphPad Software, San Diego, CA, USA).

## Results

### Behavioral analyses

**Fig. 1:**
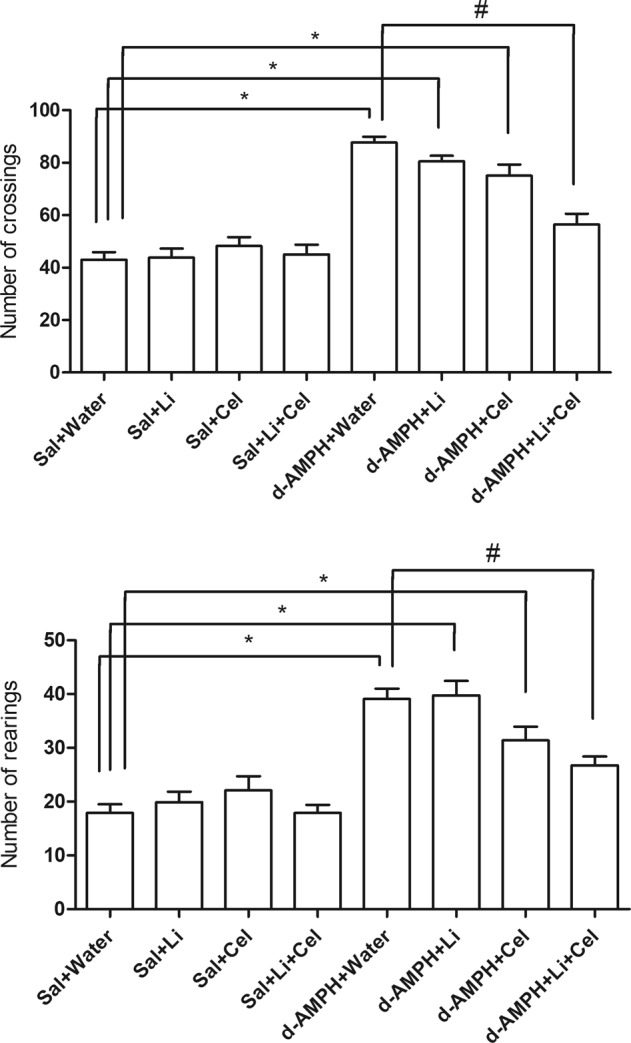
Effects of dextroamphetamine (d-AMPH) administration on the number of crossings and rearings in adult rats submitted to the animal model of mania induced by d-AMPH (*n* = 12 per group). Animals orally received lithium (Li; 24 mg/kg body weight), celecoxib (Cel; 20 mg/kg body weight), or a combination thereof. Data were analyzed by three-way analysis of variance followed by Tukey’s test when *F* was significant. Values are expressed as the mean ± standard error of the mean (arbitrary units). *p < 0.05 compared with saline (Sal) + water group. ^#^*p* < 0.05 compared with d-AMPH + water group

It was performed the open-field test to evaluate rat locomotion, expressed as the number of crossings and rearings (Fig. 1). It was observed that rats receiving d-AMPH presented a marked pattern of locomotion characterized by an increased number of crossings and rearings, as compared with the Sal + water group animals. Coadministration of Li and Cel reversed the increase in crossings and rearings induced by d-AMPH.

Data from three-way ANOVA revealed significant effects of d-AMPH administration [crossing: *F*(1, 80) = 18.84, *p* < 0.001; rearing: *F*(1, 80) = 14.38, *p* < 0.001], treatment [crossing: *F*(1, 80) = 5.50, *p* = 0.02; rearing: *F*(1, 80) = 0.08, *p* = 0.77], d-AMPH administration × treatment interaction [crossing: *F*(1, 80) = 2.44, *p* = 0.12; rearing: *F*(1, 80) = 3.63, *p* = 0.06] and d-AMPH administration × Cel plus Li interaction [crossing: *F*(1, 80) = 0.55, *p* = 0.46; rearing: *F*(1, 80) = 0.02, *p* = 0.88].

### Protein carbonyl content

**Fig. 2:**
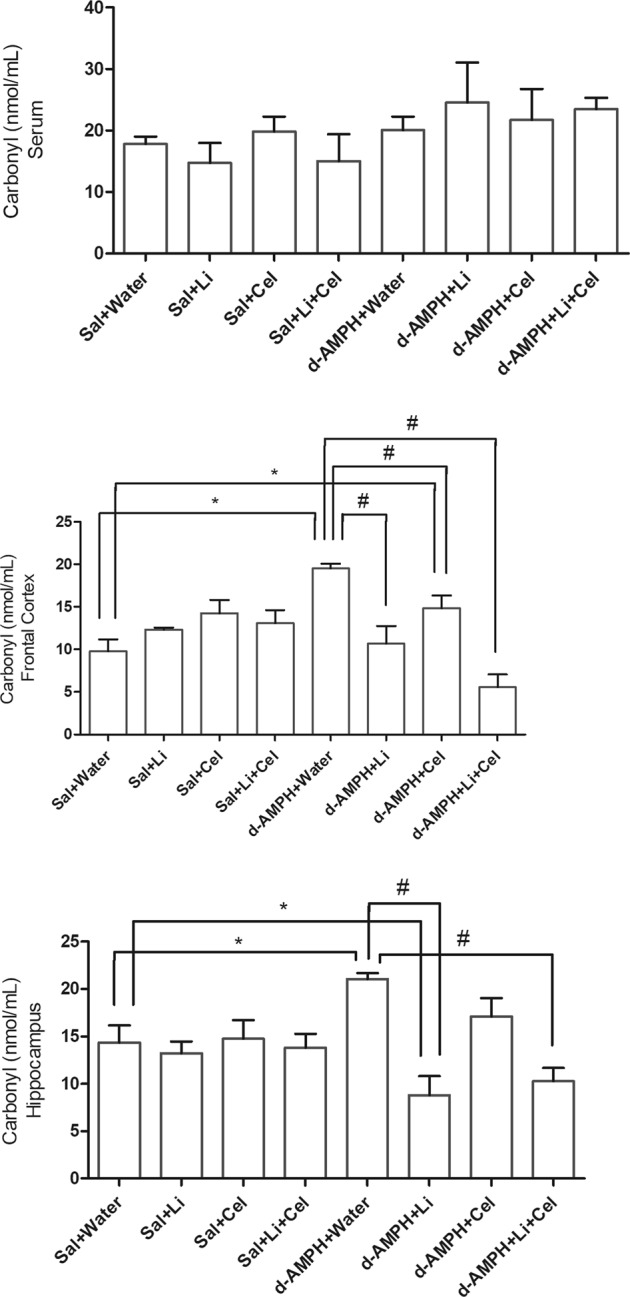
Effects of dextroamphetamine (d-AMPH) administration on the carbonyl content in serum, frontal cortex, and hippocampus of adult rats submitted to the animal model of mania induced by d-AMPH (*n* = 5 per group). Animals orally received lithium (Li; 24 mg/kg body weight), celecoxib (Cel; 20 mg/kg body weight), or a combination thereof. Data were analyzed by three-way analysis of variance followed by Tukey’s test when *F* was significant. Values are expressed as nmol/mL. *p < 0.05 compared with saline (Sal) + water group. ^#^*p* < 0.05 compared with d-AMPH + water group

Figure 2 depicts the findings on carbonyl content determination. In rat serum, no significant differences between groups were detected. However, this parameter was significantly increased in the frontal cortex and hippocampus of animals receiving water plus d-AMPH, as compared with Sal + water group. Treatment with Li or Li plus Cel reversed the increase in carbonyl content induced by d-AMPH. Also, significant decreases in the carbonyl content were detected in the frontal cortex of rats submitted to the model of mania that received Cel (d-AMPH + Cel group); however, carbonyl levels in this group remained higher than those detected in the Sal + water group animals. Administration Li plus Cel to the animals submitted to the model of mania (d-AMPH + Li + Cel group) also reversed the increase in the carbonyl content induced by d-AMPH in the hippocampus. Interestingly, a significant decrease in this parameter was detected in the hippocampus of rats receiving d-AMPH plus Li, as compared with Sal + water and d-AMPH + water groups.

Data from three-way ANOVA revealed significant effects of d-AMPH administration [frontal cortex: *F*(1, 28) = 11.91, *p* < 0.01; hippocampus: *F*(1, 30) = 0.57, *p* = 0.45; serum: *F*(1, 32) = 0.03, *p* *=* 0.86], treatment [frontal cortex: *F*(1, 28) = 20.01, *p* < 0.001; hippocampus: *F*(1, 30) = 13.91, *p* < 0.001; serum: *F*(1, 32) = 2.06, *p* *=* 0.16], d-AMPH administration × treatment interaction [frontal cortex: *F*(1, 28) = 0.92, *p* = 0.35; hippocampus: *F*(1, 30) = 1.49, *p* = 0.23; serum: *F*(1, 32) = 0.20, *p* = 0.66] and d-AMPH administration × Cel plus Li interaction [frontal cortex: *F*(1, 28) = 0.57, *p* = 0.45; hippocampus: *F*(1, 30) = 1.30, *p* = 0.26; serum: *F*(1, 32) = 0.01, *p* = 0.92].

### TBARS levels

**Fig. 3:**
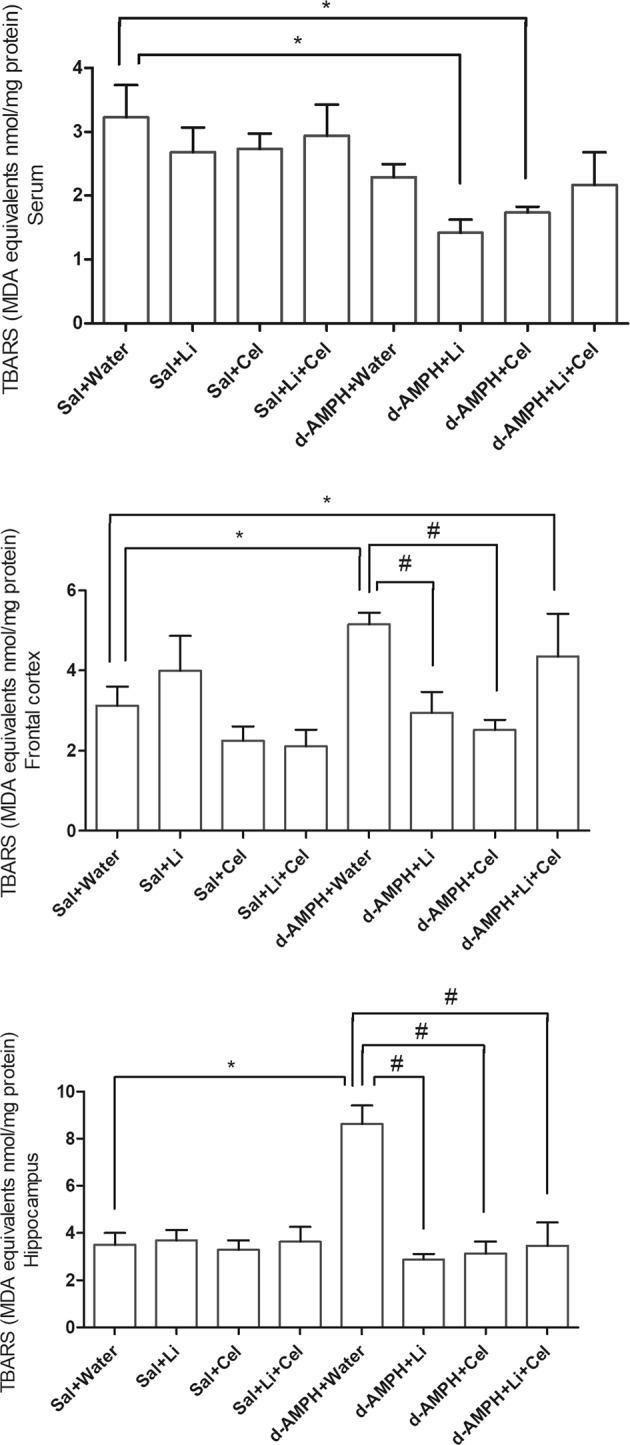
Effects of dextroamphetamine (d-AMPH) administration on the thiobarbituric acid-reactive species (TBARS), malondialdehyde (MDA), in serum, frontal cortex, and hippocampus of adult rats submitted to the animal model of mania induced by d-AMPH (*n* = 5 per group). Animals orally received lithium (Li; 24 mg/kg body weight), celecoxib (Cel; 20 mg/kg body weight), or a combination thereof. Data were analyzed by three-way analysis of variance followed by Tukey’s test when *F* was significant. Values are expressed as MDA equivalents nmol/mg protein. *p < 0.05 compared with saline (Sal) + water group. ^#^*p* < 0.05 compared with d-AMPH + water group

The next step of the present study was to evaluate the content of TBARS in serum, frontal cortex, and hippocampus samples of rats (Fig. 3). A significant decrease in this parameter was detected in animals submitted to the model of mania receiving Li or Cel, as compared with the Sal + water group. In contrast, a marked increase in this parameter was observed in the frontal cortex and hippocampus of rats only receiving water plus d-AMPH, as compared with Sal + water group. Animals submitted to the experimental model receiving Li or Cel (d-AMPH + Li or d-AMPH + Cel groups), but not Li plus Cel (d-AMPH + Li + Cel), presented a significant decrease in TBARS level detected in the frontal cortex, as compared with the rats only receiving water plus d-AMPH. Also, a significant decrease in this parameter was observed in the hippocampus of animals submitted to the model receiving Li, Cel or Li plus Cel, in comparison to the rats in similar conditions but only receiving water plus d-AMPH.

Data from three-way ANOVA revealed significant effects of d-AMPH administration [frontal cortex: *F*(1, 36) = 0.26, *p* = 0.61; hippocampus: *F*(1, 38) = 7.26, *p* = 0.01; serum: *F*(1, 36) = 1.20, *p* *=* 0.66], treatment [frontal cortex: *F*(1, 36) = 1.11, *p* = 0.30; hippocampus: *F*(1, 38) = 11.92, *p* < 0.01; serum: *F*(1, 36) = 0.01, *p* *=* 0.91], d-AMPH administration × treatment interaction [frontal cortex: *F*(1, 36) = 4.86, *p* = 0.034; hippocampus: *F*(1, 38) = 13.05, *p* < 0.001; serum: *F*(1, 36) = 4.67, *p* = 0.03] and d-AMPH administration × Cel plus Li interaction [frontal cortex: *F*(1, 36) = 11.70, *p* < 0.01; hippocampus: *F*(1, 38) = 11.82, *p* < 0.01; serum: *F*(1, 36) = 0.33, *p* = 0.56].

### CAT enzyme activity

**Fig. 4:**
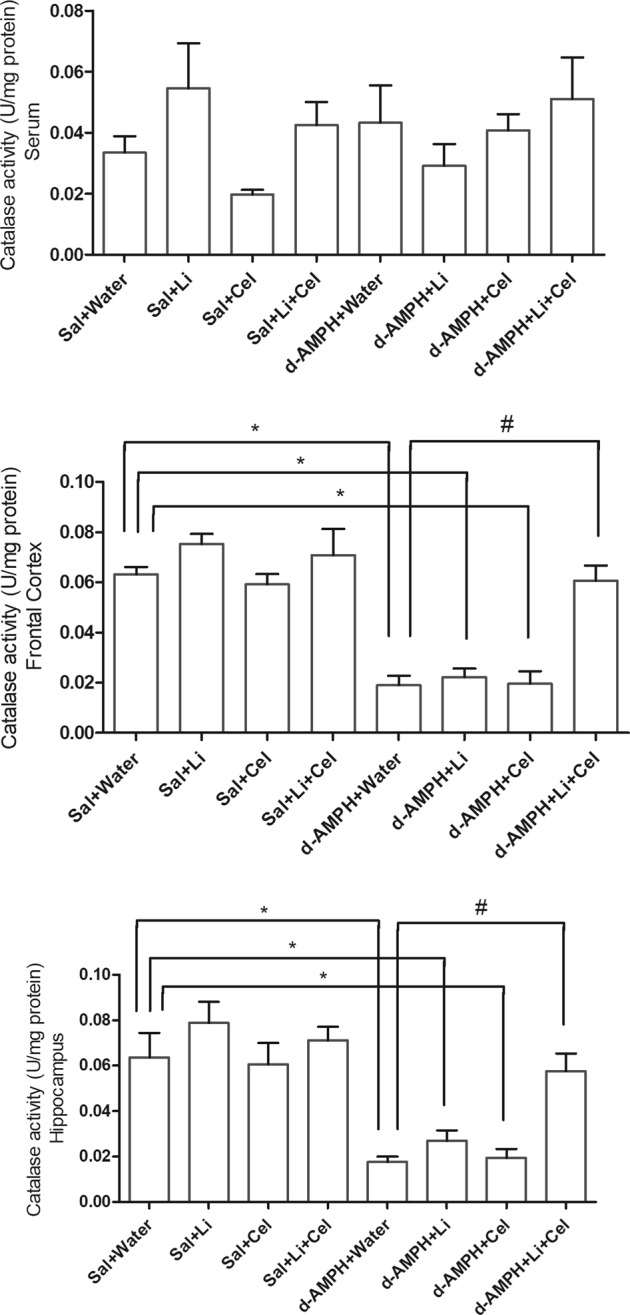
Effects of dextroamphetamine (d-AMPH) administration on the activity of catalase enzyme in serum, frontal cortex, and hippocampus of adult rats submitted to the animal model of mania induced by d-AMPH (*n* = 5 per group). Animals orally received lithium (Li; 24 mg/kg body weight), celecoxib (Cel; 20 mg/kg body weight), or a combination thereof. Data were analyzed by three-way analysis of variance followed by Tukey’s test when *F* was significant. Values are expressed as U/mg protein. *p < 0.05 compared with saline (Sal) + water group. #*p* < 0.05 compared with d-AMPH + water group

Another important marker of oxidative stress evaluated in the present study was the activity of the CAT enzyme (Fig. 4). In rat serum, it was detected no significant differences in this parameter between groups. Nevertheless, a significant decrease in CAT activity was observed in the frontal cortex and hippocampus of animals submitted to the d-AMPH administration and receiving water, Li or Cel, as compared with the Sal + water group. A significant recovery of the enzyme activity in these cerebral structures was only detected when the rats submitted to the experimental model received Li plus Cel, in comparison with the animals receiving water plus d-AMPH.

Data from three-way ANOVA revealed significant effects of d-AMPH administration [frontal cortex: *F*(1, 31) = 10.86, *p* < 0.01; hippocampus: *F*(1, 27) = 4.92, *p* = 0.03; serum: *F*(1, 32) = 2.86, *p* *=* 0.10], treatment [frontal cortex: *F*(1, 31) = 2.03, *p* = 0.16; hippocampus: *F*(1, 27) = 1.26, *p* = 0.27; serum: *F*(1, 32) = 3.16, *p* *=* 0.085], d-AMPH administration × treatment interaction [frontal cortex: *F*(1, 31) = 6.72, *p* = 0.014; hippocampus: *F*(1, 27) = 1.54, *p* = 0.22; serum: *F*(1, 32) = 0.96, *p* *=* 0.33] and d-AMPH administration × Cel plus Li interaction [frontal cortex: *F*(1, 31) = 7.08, *p* = 0.012; hippocampus: *F*(1, 27) = 2.96, *p* = 0.097; serum: *F*(1, 32) = 0.73, *p* *=* 0.40].

### GPx enzyme activity

**Fig. 5:**
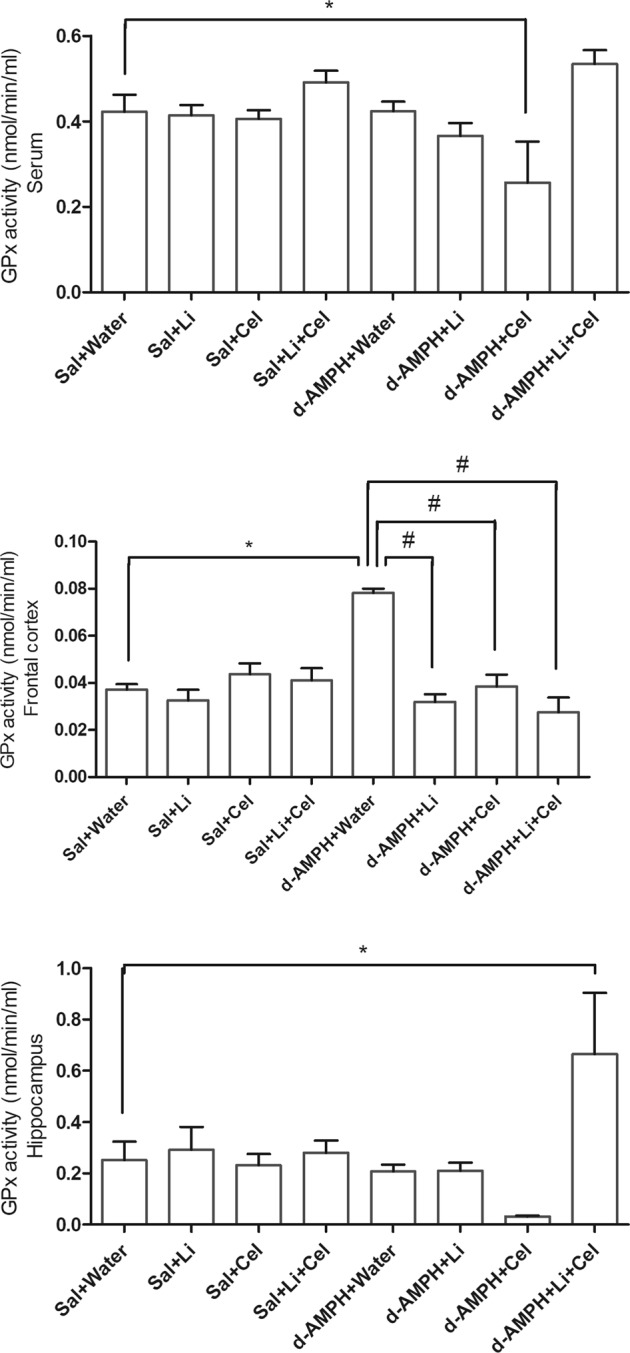
Effects of dextroamphetamine (d-AMPH) administration on the activity of glutathione peroxidase (GPx) enzyme in serum, frontal cortex, and hippocampus of adult rats submitted to the animal model of mania induced by d-AMPH (*n* = 5 per group). Animals orally received lithium (Li; 24 mg/kg body weight), celecoxib (Cel; 20 mg/kg body weight), or a combination thereof. Data were analyzed by three-way analysis of variance followed by Tukey’s test when *F* was significant. Values are expressed as nmol/min/mL (nmol min^−1^ mL^−1^). *p < 0.05 compared with saline (Sal) + water group. ^#^*p* < 0.05 compared with d-AMPH + water group

The last parameter investigated in the present work was the activity of GPx enzyme (Fig. 5). In rat serum, a significant decrease in this parameter was detected in animals receiving d-AMPH plus Cel, as compared with Sal + water group. In contrast, a marked increase in the GPx activity was observed in the frontal cortex of rats only receiving water plus d-AMPH and in the hippocampus of animals submitted to the d-AMPH administration and treated with Li plus Cel. In the frontal cortex, a significant decrease of this enzyme activity was detected in rats submitted to the d-AMPH administration and treated with Li, Cel or Li plus Cel, in comparison with animals only receiving water plus d-AMPH.

Data from three-way ANOVA revealed significant effects of d-AMPH administration [frontal cortex: *F*(1, 32) = 22.15, *p* < 0.001; hippocampus: *F*(1, 30) = 0.39, *p* = 0.53; serum: *F*(1, 36) = 0.22, *p* = 0.64], treatment [frontal cortex: *F*(1, 32) = 15.94, *p* < 0.001; hippocampus: *F*(1, 30) = 0.16, *p* = 0.69; serum: *F*(1, 36) = 1.23, *p* = 0.27], d-AMPH administration × treatment interaction [frontal cortex: *F*(1, 32) = 8.96, *p* < 0.01; hippocampus: *F*(1, 30) = 2.38, *p* = 0.13; serum: *F*(1, 36) = 11.13, *p* *<* 0.01] and d-AMPH administration × Cel plus Li interaction [frontal cortex: *F*(1, 32) = 7.05, *p* = 0.012; hippocampus: *F*(1, 30) = 0.11, *p* = 0.74; serum: *F*(1, 36) = 3.52, *p* = 0.07].

## Discussion

In the present study, d-AMPH administration elicited hyperlocomotion in rats, which correlates with manic behavior or hyperactivity in BD patients^9^. Following the data provided by behavioral analysis, previous evidence showed that the administration of d-AMPH induces hyperactivity in rats^29,30^. Furthermore, studies have suggested 47.5 mg/kg body weight as the therapeutic dose of Li^7,30^. It was observed here that the administration of such drug at a lower dose (24 mg/kg) did not mitigate the hyperlocomotion induced by d-AMPH in the animals. However, this dose was used to investigate the putative therapeutic potential of Li and Cel coadministration, owing to the need for potential reduction of Li side effects (at 47.5 mg/kg).

The administration of Cel elicited no significant effect on the hyperactivity induced by d-AMPH in rats, but the administration of this cyclooxygenase inhibitor (20 mg/kg) plus Li (24 mg/kg) significantly abrogated the effect of d-AMPH. Recently, a study showed that coadministration of these drugs in the same doses also was implicated in a decrease in the hyperlocomotion of rats induced by d-AMPH^31^. Interestingly, it was reported that the administration of Cel during two weeks was showed to reverse the hyperactivity observed in bulbectomized animals^32^. In addition, the role of certain anti-inflammatory drugs as potential adjuvants for BD therapy has been investigated. A placebo-controlled, randomized trial showed that patients affected by such disorder receiving Cel during 1 week presented a significant improvement in their depressive symptoms, as compared with the placebo group^19^. Cyclooxygenase inhibitors were also implicated in the modulation of behaviors depending on dopaminergic neurotransmission, as described in the study headed by Ross using indomethacin in order to regulate the hyperactivity induced by d-AMPH (1 mg/kg)^33^.

Evidence regarding the pharmacological action of Cel on the dopaminergic neuronal pathway is scarce yet. Nevertheless, inhibition of cyclooxygenase-2 by this drug was implicated in the regulation of dopamine levels, as described in a schizophrenia model induced by intracranial injection of epidermal growth factor in rats^34^. Moreover, Cel was reported to mitigate the degeneration of dopaminergic neurons induced by intrastriatal injection of 6-hydroxydopamine—an effect partly mediated by inhibition of microglia activity^35^. Cel administration (20 mg/kg) also alleviated the behavioral abnormalities, dopaminergic dysfunction, and produced a decrease in the levels of activated microglia in the brain of young rats submitted to the model of neuroinflammation induced by lipopolysaccharide^36^. Thus, here was speculate that Cel could regulate the activity of dopaminergic signaling underlying the hyperactivity observed in rats receiving d-AMPH, whereas the administration of Li could enhance this modulation. Whether this central pharmacological action of Cel involves the abrogation of microglia activation potentially induced by d-AMPH is an issue that requires further investigation.

Indeed, there is evidence indicating that the decrease in dopamine synthesis and increase in intracellular levels of such neurotransmitter could counteract and induce the activation of microglia in the striatum of animals, respectively^36,37^, which provides a potential link between the administration of d-AMPH and inflammatory response in the dopaminergic model of mania using this drug. Besides, aberrant levels of proinflammatory mediators termed cytokines has been reported in the serum of bipolar patients^38^. As amphetamines elicit persistent damage to dopaminergic neurons, the subsequent microglia activation could, in turn, collaborate to the release of certain cytokines^39^. Thus, it cannot be ruled out that Li and Cel mutually act to mitigate the proinflammatory state reported in animal models of mania and bipolar patients, possibly through the inhibition of cyclooxygenase-2 enzyme and regulation of cytokine levels^40,41^.

Oxidative stress is also proposed as a crucial factor for the cytotoxicity elicited by amphetamines^42^. The increase in the level of reactive oxygen species induced by d-AMPH may lead to the release of cytochrome *c* and mitochondrial DNA into the cytosol, subsequently resulting in cellular damage and inflammatory response^43,44^. Indeed, it was observed that the administration of d-AMPH induced oxidative damage to lipids (evaluated as TBARS levels) and proteins (as carbonyl content), whereas decreased the activity of CAT and GPx antioxidant enzymes in rat brain. It is worthy to note that Frey et al.^8^ also detected an increased level of TBARS in the hippocampus and frontal cortex samples of rats submitted to the dopaminergic model of mania, which was consistent to oxidative stress in the brain of these animals. As oxidative stress is an imbalance between the generation of reactive oxygen species and the biological antioxidant defenses^45^, the present study suggests that lipid and protein oxidative damage elicited by d-AMPH could in part be a consequence of the CAT and GPx inhibition.

Administration of Cel significantly reversed the increased TBARS level induced by d-AMPH in the brain of rats, whereas partially reversed the increased carbonyl content in the frontal cortex. Such finding corroborates by a study showing that the administration of Cel can mitigate oxidative lipid damage^46^. However, the present study was the first to indicate that the administration of Cel significantly prevents the protein carbonylation induced by d-AMPH. Potential mechanisms involved in the antioxidant and anti-inflammatory activity of Cel include a decrease in the synthesis of cytokines and the release of excitatory amino acids, modulation of the inducible nitric oxide synthase (EC 1.14.13.39) pathway and decrease in the generation of hydroxyl radical (•OH)^47^.

On the other hand, the decreased activity of CAT in rats receiving d-AMPH was not affected by the Cel administration. Accordingly, Venugopal and Prakash^48^ showed a dose-dependent effect of Cel on the CAT activity in an experimental model of neuroinflammation: 10 mg/kg was not able to alter the activity of the enzyme, whereas a significantly increased CAT activity was detected in response to a higher dose of the drug (50 mg/kg). In this scenario, the dose of Cel used in the present study (20 mg/kg) was unable to modify the activity of this enzyme. However, Cel (20 mg/kg) modulated the activity of GPx in animals submitted to the model of mania, whereas it was reported that the coadministration of Cel and cisplatin or tumoral necrosis factor (TNF)-α decreased the levels of GPx^49^. Overall, these studies and the findings of the present study indicate that Cel regulation of CAT and GPx activities are part of antioxidant action mechanism of the drug.

Under previous work, administration of Li reversed the oxidative lipid damage in the brain of rats submitted to the model of mania^8^. Such drug was also implicated in decreased carbonyl content in these animals. This finding seems to agree with other preclinical work, which showed that the administration of Li (47.5 mg/kg) reversed the increase in the carbonyl content induced by methamphetamine^50^. Furthermore, Li was showed to prevent the mitochondrial electron transport chain complexes from damage elicited by d-AMPH^29^. Taking into account that reactive oxygen species are mainly generated in mitochondria^51^, it could be suggested that Li exerts the aforementioned antioxidant roles partly through the modulation of the mitochondrial respiratory chain activity. Indeed, neuroprotective activity was also assigned to this mood stabilizer, collaborating to its therapeutic properties^52^.

It was also observed that the administration of Li abrogated the alteration in the activity of GPx, but not in CAT, in animals receiving d-AMPH. This finding partly agrees with the paper performed by Macêdo and colleagues^53^, which showed that Li at the therapeutic dose (47.5 mg/kg) significantly regulated GPx activity in the presence of lisdexamphetamine. In contrast to the present work, Li at the therapeutic range significantly reversed the modification in the activity of CAT elicited by d-AMPH^8^. Although the activity of these enzymes was not linear in most of the samples, there was a linear association between CAT and GPx activities, which indicates that each cerebral structure could present a typical pattern of antioxidant enzyme activity in response to d-AMPH.

Coadministration of Li and Cel counteracted the increase in cerebral carbonyl content and TBARS level in hippocampus of animals receiving d-AMPH. In contrast to the single administration of each drug, only the combined approach was able to mitigate the alterations in the antioxidant enzymes detected in the model of mania. According to the described elsewhere, both Li and Cel present antioxidant properties^54,55^. Concerning the combined administration of these drugs, Phelan and coworkers^56^ reported that Li presents a possible pharmacokinetic interaction with Cel. More specifically, these researchers showed that the plasma level of Li increased by 99% when this drug was administered with Cel^56^, which could be related to the ability of Cel to decrease Li renal clearance^57^. Thus, Cel could increase the plasma levels of the mood stabilizer, although the levels of Li in rat serum were not measured in the present study.

On the other hand, it cannot be ruled out that both drugs could also act synergistically. Indeed, Cel and Li exhibit either anti-inflammatory and antioxidant activities^58,59^, which could be of interest for BD, as increased levels of inflammation markers (e.g., interleukin (IL)-1β, IL-6, TNF-α, and soluble receptors for IL-2 and TNF) were detected in the serum of bipolar patients^60^. These findings are consistent with the inflammatory hypothesis for BD manic symptoms^61^. It is worthy to note that proinflammatory cytokines were implicated in the depletion of 5-hydroxytryptamine in the central nervous system^58^. In contrast, Cel per se was also associated with a moderate antidepressant activity^19,62,63^, which could be in part related to the ability of the drug to enhance 5-hydroxytryptamine release in the medial prefrontal cortex^64^.

Therefore, regarding present findings and all the studies above, it is possible that Li and Cel act in order to stabilize the moods and prevent the oxidative damage by one or more of the following mechanisms: (1) regulation of the dopamine levels through inhibition of cyclooxygenase-2 triggered by Cel; (2) inhibition of microglia activity via a decrease in dopamine synthesis in the striatum; (3) the decrease in the synthesis of cytokines and the release of excitatory amino acids, as well as modulation of the inducible nitric oxide synthase pathway (especially Cel); (4) modulation of the electron transport chain activity, preventing the mitochondrial complexes from the damage elicited by d-AMPH (especially Li); (5) a possible pharmacokinetic interaction between Li and Cel, characterized by a decrease in the renal clearance of the mood stabilizer induced by the anti-inflammatory drug; (6) since proinflammatory cytokines were implicated in decreased 5-hydroxytryptamine levels in the brain, Cel could counteract this depletion, in part enhancing the release of the neurotransmitter in the medial prefrontal cortex.

In summary, hyperlocomotion and significant alteration in oxidative stress biomarkers were detected in the cerebral structures of rats submitted to a model of mania induced by d-AMPH, corroborating with previous observations that such chemical species are involved in the pathophysiology of BD. Administration of Li plus Cel can mitigate these imbalances, rendering this combined approach a potential novel therapeutic option to the management of BD. Additional preclinical research is also needed to explore the safety of this promising combined therapy in the context of BD.

### Limitations of the study

Measurements of Li serum levels were not performed. Besides, one of the most critical advantages of preclinical research in Psychiatry reflects the possibility to establish an engagement of a specific neurotherapeutic target. However, current findings are still correlational and do not provide this level of evidence, which highlights the importance of further research in the field. Despite these drawbacks, the principal aims of the present study were to provide additional evidence to support further preclinical and clinical research on the Li and Cel coadministration, as well as provide a drug repositioning strategy based on the anti-inflammatory drug.
